# The *Bcl*-*2* homologue *Buffy* rescues *α*-*synuclein*-induced Parkinson disease-like phenotypes in Drosophila

**DOI:** 10.1186/s12868-016-0261-z

**Published:** 2016-05-18

**Authors:** P. Githure M’Angale, Brian E. Staveley

**Affiliations:** Department of Biology, Memorial University of Newfoundland, St. John’s, NL A1B 3X9 Canada

## Abstract

**Background:**

In contrast to the complexity found in mammals, only two *Bcl*-*2* family genes have been found in *Drosophila melanogaster* including the pro-cell survival, human *Bok*-related orthologue, *Buffy.* The directed expression of *α*-*synuclein*, the first gene identified to contribute to inherited forms of Parkinson disease (PD), in the dopaminergic neurons (DA) of flies has provided a robust and well-studied Drosophila model of PD complete with the loss of neurons and accompanying motor defects. To more fully understand the biological basis of *Bcl*-*2* genes in PD, we altered the expression of *Buffy* in the dopamine producing neurons with and without the expression of *α*-*synuclein,* and in the developing neuron-rich eye.

**Results:**

To alter the expression of *Buffy* in the dopaminergic neurons of Drosophila, the *Ddc*-*Gal4* transgene was used. The directed expression of *Buffy* in the dopamine producing neurons resulted in flies with increased climbing ability and enhanced survival, while the inhibition of *Buffy* in the dopaminergic neurons reduced climbing ability over time prematurely, similar to the phenotype observed in the *α*-*synuclein*-induced Drosophila model of PD. Subsequently, the expression of *Buffy* was altered in the *α*-*synuclein*-induced Drosophila model of PD. Analysis revealed that *Buffy* acted to rescue the associated loss of locomotor ability observed in the *α*-*synuclein*-induced model of PD, while Buffy RNA interference resulted in an enhanced *α*-*synuclein*-induced loss of climbing ability. In complementary experiments the overexpression of *Buffy* in the developing eye suppressed the mild rough eye phenotype that results from *Gal4* expression and from *α*-*synuclein* expression. When Buffy is inhibited the roughened eye phenotype is enhanced.

**Conclusions:**

The inhibition of Buffy in DA neurons produces a novel model of PD in Drosophila. The directed expression of *Buffy* in DA neurons provide protection and counteracts the *α*-*synuclein*-induced Parkinson disease-like phenotypes. Taken all together this demonstrates a role for *Buffy*, a *Bcl*-*2* pro-cell survival gene, in neuroprotection.

## Background

Parkinson disease (PD) is the most common human movement disorder and the second most common neurodegenerative disease; afflicting about 1–2 % of the population over 50 years of age [1, 2]. PD is strongly associated with the selective and profound loss of dopaminergic (DA) neurons to result in marked clinical features which include muscle rigidity, resting tremors, postural instability, bradykinesia as well as non-motoric symptoms like autonomic, cognitive and psychiatric problems [2]. The neuropathological hallmarks exhibited by PD patients include the presence of Lewy Bodies (LB) and Lewy Neurites (LN) in surviving neurons. This is due to the loss of DA neurons in the *substantia nigra pars compacta* (*SNpc*) region of the brain, coupled with the presence of eosinophilic, intracytoplasmic proteinaceous inclusions comprised of the α-synuclein and ubiquitin proteins, among others [2–4]. This unusual protein accumulation is believed to lead to cellular toxicity and, eventually, the PD pathogenesis. Other associated pathological mechanisms include aberrant protein aggregation and mitochondrial damage [5–7]. Although the majority of PD cases are considered to be sporadic, familial forms have been documented and much has been discovered through study of the associated gene loci in model organisms [8–10]. PARK1/4 was the first gene associated with PD to be identified [3], and it encodes for a small soluble protein of unknown function predominantly found in neural tissues [3, 8, 11]. The first Drosophila model of PD utilized a human α-synuclein transgene to induce the PD-like symptoms [12]. The success of this model is its ability to recapitulate features of human PD such as (1) age-dependent loss in locomotor function (2) LB-like inclusions and (3) age-dependent loss of DA neurons; and therefore has found wide use for studying the molecular basis of α-synuclein-induced neurodegeneration [12–19]. The utilization of the UAS/GAL4 spatio-temporal expression system [20], and the availability of a plethora of promoters or enhancers of which TH-Gal4, elav-Gal4 and Ddc-Gal4 are employed in modelling PD in flies, makes Drosophila a powerful model organism [12–19]. Mitochondrial dysfunction due to the accumulation of α-synuclein has been implicated as one of the mechanisms leading to PD [21–24]. The association of α-synuclein with components of the mitochondria is thought to lead to oxidative stress, apoptosis, autophagy and the eventual neurodegeneration.

The *Bcl*-*2* family of genes are key regulators of cell death and survival in animals and are functionally composed of proapoptotic and pro-cell survival (antiapoptotic) members [25–28]. The pro-survival proteins protect the mitochondria in part, from disruption by the proapoptotic proteins [26, 29–32]. In mammals, the antiapoptotic members possess four Bcl-2 homology (BH) domains—BH1, BH2, BH3 and BH4—and include Bcl-2, BclX_L_, Mcl-1 among others. The proapoptotic members, Bax, Bak and Bok, have three BH domains: BH1, BH2 and BH3. A BH3-only domain class of proapoptotic proteins is present and includes Bid, Bim, Bad, Bik, Hrk, Noxa and Puma [33–35]. The multi-domain proapoptotic proteins are required for mitochondrial outer membrane (MOM) permeabilization and the subsequent release of apoptogenic factors into the cytosol [36–39]. As thus, the antiapoptotic members guard the mitochondria and stop the release of a plethora of death causing molecules that initiate apoptosis.

The *Bcl*-*2* family of proteins are thought to be the “guardians” of the mitochondria, involved in the life and death decisions at the cellular level by initiating mitochondrial remodelling, mitochondrial outer membrane permeabilization and the release of apoptotic factors from the mitochondria [40]. This delicate balance is maintained by the activity of the pro-survival and anti-survival members of the protein family. Many of the apoptotic pathway proteins that participate in the intrinsic and extrinsic cell death pathways have been identified in Drosophila [41–43]. The *Bcl*-*2* family member homologues in Drosophila are limited to the single pro-cell survival *Buffy* and the sole pro-cell death *Debcl* [44–48]. These two proteins share a strong similarity within their domains and with the mammalian pore-forming proapoptotic member Bok/Mtd.

In previous studies, the overexpression of *Buffy* has been shown to suppress *Pink1* mutant phenotypes [49] and suggest a role for this protein (1) in interacting with the Pink1 protein and other mitochondrial proteins or (2) in a pathway that regulates mitochondrial function and integrity. Studies show that *Buffy* has little involvement in cell death during development [50], though it has a role in regulating cell death that occurs in response to external stimuli and a role in the mitochondrial pathway for the activation of cell death during Drosophila oogenesis [51], all which point to an important role for this protein in aspects of cell death. Indeed, early studies have demonstrated that *Buffy* plays roles in both anti- and pro-survival [52, 53] depending upon the stimuli.

A direct role for the Bcl-2 proteins in mitochondrial dynamics has been shown in the activation of cell death in *Drosophila melanogaster* during mid-oogenesis [51] and in the *Pink1* loss-of-function Parkinson disease model [49]. The predicted role of the mitochondria in PD pathogenesis makes the *α*-*synuclein*-induced model of PD [12] a very attractive model for the investigation of the role of Bcl-2 proteins. First we examine the effects of increasing and decreasing Buffy activity in DA neurons and, secondly, we investigate the potential suppression of the *α*-*synuclein*-induced PD phenotypes by the overexpression of the pro-survival Bcl-2 homologue *Buffy*.

## Results

### *Buffy* is similar to the human proapoptotic *Bok*

**Fig. 1:**
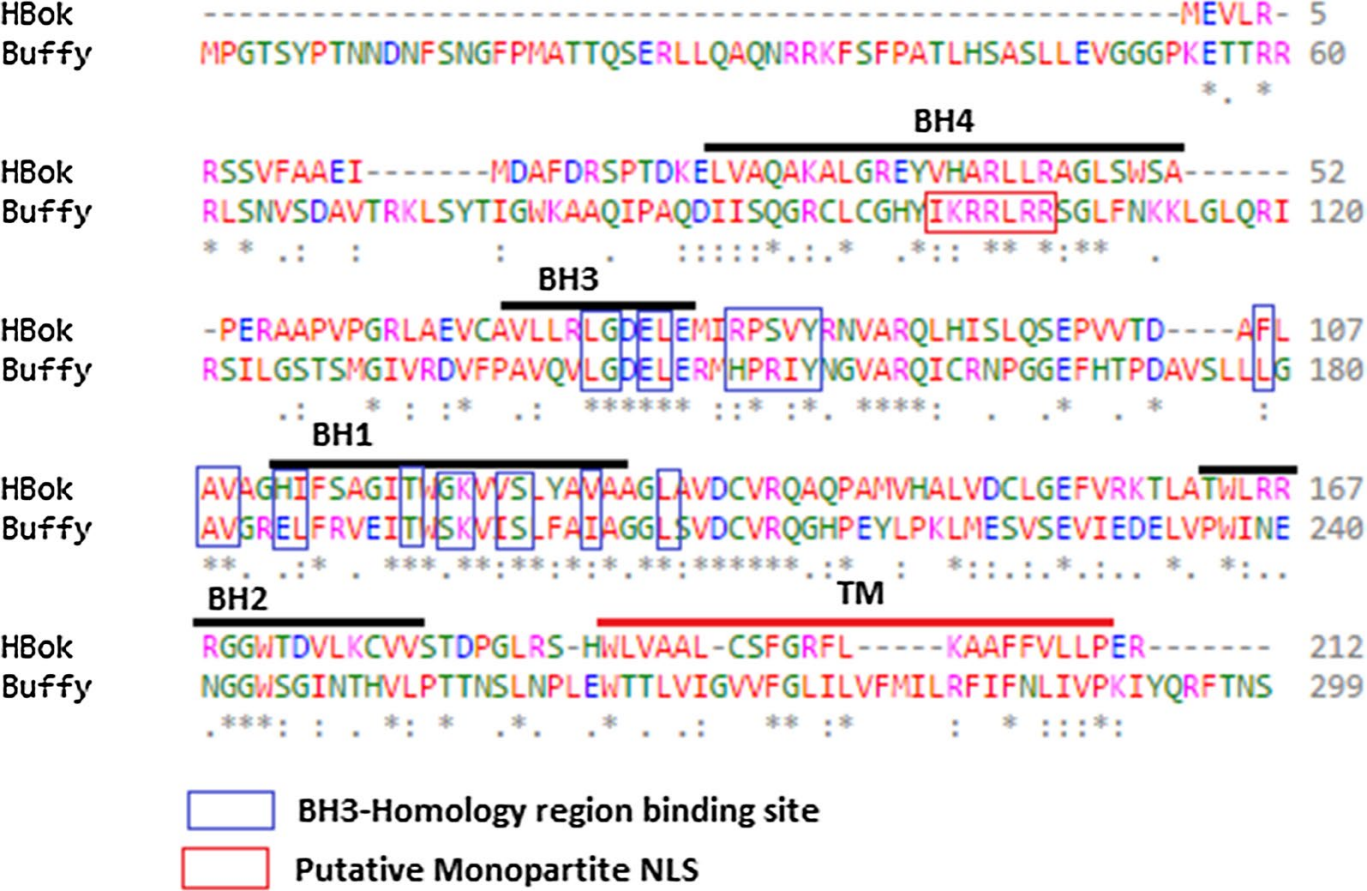
Buffy is closely related to human Bcl-2 ovarian killer (Bok). When Buffy protein is aligned with human Bok, the Bcl-2 homology (BH) domains show strong conservation. Clustal Omega multiple sequence alignment [64, 65] of *Drosophila melanogaster* Buffy protein (*Drosophila melanogaster* NP_523702.1) with the human Bok (*Homo sapiens* NP_115904.1) showing the highlighted conserved BH domains, the BH3-homology region binding site, and the TM (trans-membrane) helices. Buffy possesses a monopartite basically charged nuclear localisation signal (NLS) region. The domains were identified using NCBI Conserved Domain Database Search (CDD) [66]. “*Asterisks*” indicate the residues that are identical, “*colon*” indicate the conserved substitutions, “*dot*” indicate the semi-conserved substitutions. *Colours* show the chemical nature of amino acids: *red* is small hydrophobic (including aromatic), *blue* is acidic, *magenta* is basic, and *green* is basic with hydroxyl or amine groups

Bioinformatic analysis of the protein sequences encoded by the *Buffy* and *Bok* genes reveal 33 % identity. The Buffy protein consists of 299 amino acids and reveals the presence of the BH1, BH2, BH3, BH4 and TM domains (Fig. 1). The Eukaryotic Linear Motif (ELM) resource search for functional sites indicates the presence of a monopartite variant of a basically charged NLS between amino acids 101 and 106. There is a number of BH3-homology region binding sites in the central region of the protein. Bok has 212 amino acids and similarly shows the presence of the BH1, BH2, BH3, and BH4 domains. Although, the two proteins are determined to be antiapoptotic and proapoptotic respectively, both show the presence of a BH4 domain, the homology domain that is associated with pro-survival proteins.

### Loss of *Buffy* decreases lifespan and climbing ability

**Fig. 2:**
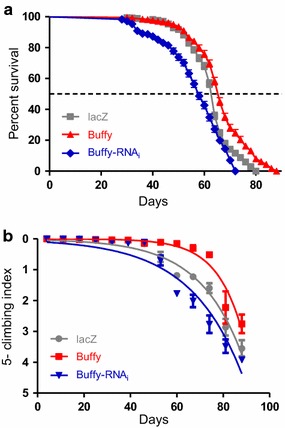
*Buffy* alters lifespan and climbing ability when mis-expressed in DA neurons. **a** Directed expression of *UAS*-*Buffy* in the DA neurons driven by *Ddc*-*Gal4* results in increased survival compared to the controls overexpressing *UAS*-*lacZ*, while inhibition via *Buffy*-*RNAi* results in reduced survival. The genotypes are *UAS*-*lacZ/Ddc*-*Gal4; UAS*-*Buffy/Ddc*-*Gal4;* and *UAS*-*Buffy*-*RNAi/Ddc*-*Gal4.* Longevity is shown as percent survival (P < 0.01, determined by log-rank and *n* ≥ 200). **b** Directed expression of *UAS*-*Buffy* results in increased climbing ability as determined by non-linear fitting of the climbing curves and comparing at 95 % confidence intervals. Inhibition by *Buffy*-*RNAi* decreased the locomotor ability when expressed in dopaminergic neurons. The genotypes are *UAS*-*lacZ/Ddc*-*Gal4; UAS*-*Buffy/Ddc*-*Gal4;* and *UAS*-*Buffy*-*RNAi/Ddc*-*Gal4. Error bars* indicate the standard error of the mean (SEM), *asterisks* represents statistically significant result and *n* = 50

When *Buffy* is inhibited in the DA neurons by RNA interference, the resulting flies have a shortened lifespan and impaired climbing ability. The median lifespan for these flies is 58 days compared to 64 days for the controls (Fig. 2a). The nonlinear fitting of the climbing curves resulted in a slope gradient that was significantly different at 95 % confidence interval (Fig. 2b). This suggests that Buffy is required for the normal functioning of DA neurons and inhibition in DA neurons confers a disadvantage by reducing lifespan and impairing the locomotor ability of these flies.

### *Buffy* increases lifespan and climbing ability when overexpressed in DA neurons

When *Buffy* is overexpressed in DA neurons, the survival parameters of these flies differ slightly (Fig. 2a), with *Buffy*-overexpressing flies having a median lifespan of 68 days compared to 64 days for the controls. The overexpression of *Buffy* in DA neurons led to an increased climbing ability as indicated by the nonlinear fitting of the curve with 95 % CI (Fig. 2b). This suggests that *Buffy* improves longevity and markedly improves climbing ability in Drosophila when expressed in DA neurons to improve the general healthspan of these flies.

### Inhibition of *Buffy* enhances the *α*-*synuclein*-induced phenotypes

**Fig. 3:**
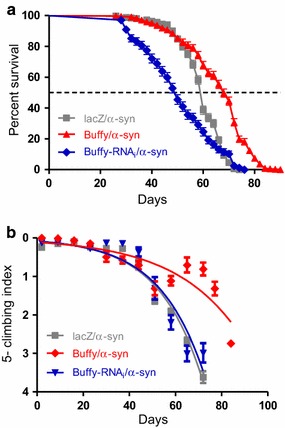
*Buffy* rescues the *α*-*synuclein*-induced phenotypes of decreased lifespan and climbing ability. **a** Directed overexpression of *Buffy* in the DA neurons increase longevity whereas flies with *Buffy* loss-of-function show a decline in lifespan. Genotypes are *UAS*-*α*-*synuclein, Ddc*-*Gal4/UAS*-*lacZ; UAS*-*α*-*synuclein, Ddc*-*Gal4/UAS*-*Buffy;* and *UAS*-*α*-*synuclein, Ddc*-*Gal4/UAS*-*Buffy*-*RNAi.* Longevity is shown as percent survival (P < 0.01, determined by log-rank and *n* ≥ 200). **b** The co-expression of *Buffy* in the *α*-*synuclein* model of PD rescued the age-dependent loss in climbing ability. The directed overexpression of *Buffy* in the DA neurons remarkably increased the climbing ability over time compared to the control, while the suppression of *Buffy* resulted in flies that climbed similar to the control. The genotypes are *UAS*-*α*-*synuclein; Ddc*-*Gal4/UAS*-*lacZ, UAS*-*α*-*synuclein; Ddc*-*Gal4/UAS*-*Buffy,* and *UAS*-*α*-*synuclein; Ddc*-*Gal4/UAS*-*Buffy*-*RNAi.* Analysis of the climbing curves and significance was determined by comparing the 95 % confidence intervals. *Error bars* indicate the SEM, *asterisks* represents statistically significant result and *n* = 50

The inhibition of *Buffy* by RNA interference when co-expressed with *α*-*synuclein*, under the directions of *Ddc*-*Gal4*, results in short-lived flies with strongly impaired locomotor function. The median lifespan of the *α*-*synuclein*-expressing control flies was 60 days, while that of those co-expressing the *Buffy*-*RNAi* inhibitory transgene along with *α*-*synuclein* was 50 days (Fig. 3a). The climbing ability of these flies was more impaired than those expressing *α*-*synuclein* alone, as indicated by the nonlinear fitting of the climbing curves (Fig. 3b).

### Overexpression of *Buffy* in DA neurons rescues the *α*-*synuclein*-induced loss of climbing ability

The overexpression of *Buffy* in DA neurons expressing *α*-*synuclein* results in an increased median lifespan of 68 days, compared to 60 days for the control flies (Fig. 3a). The climbing curves indicate that there was a significant improvement in the climbing ability of the flies when *Buffy* was overexpressed (Fig. 3b) and thus, suppressing the phenotypes observed when *α*-*synuclein* is expressed in DA neurons. This suggests that the overexpression of *Buffy* confers protection to DA neurons, as a result of the expression of *α*-*synuclein*.

### Overexpression of *Buffy* suppresses the *α*-*synuclein*-induced developmental defects in the eye

**Fig. 4:**
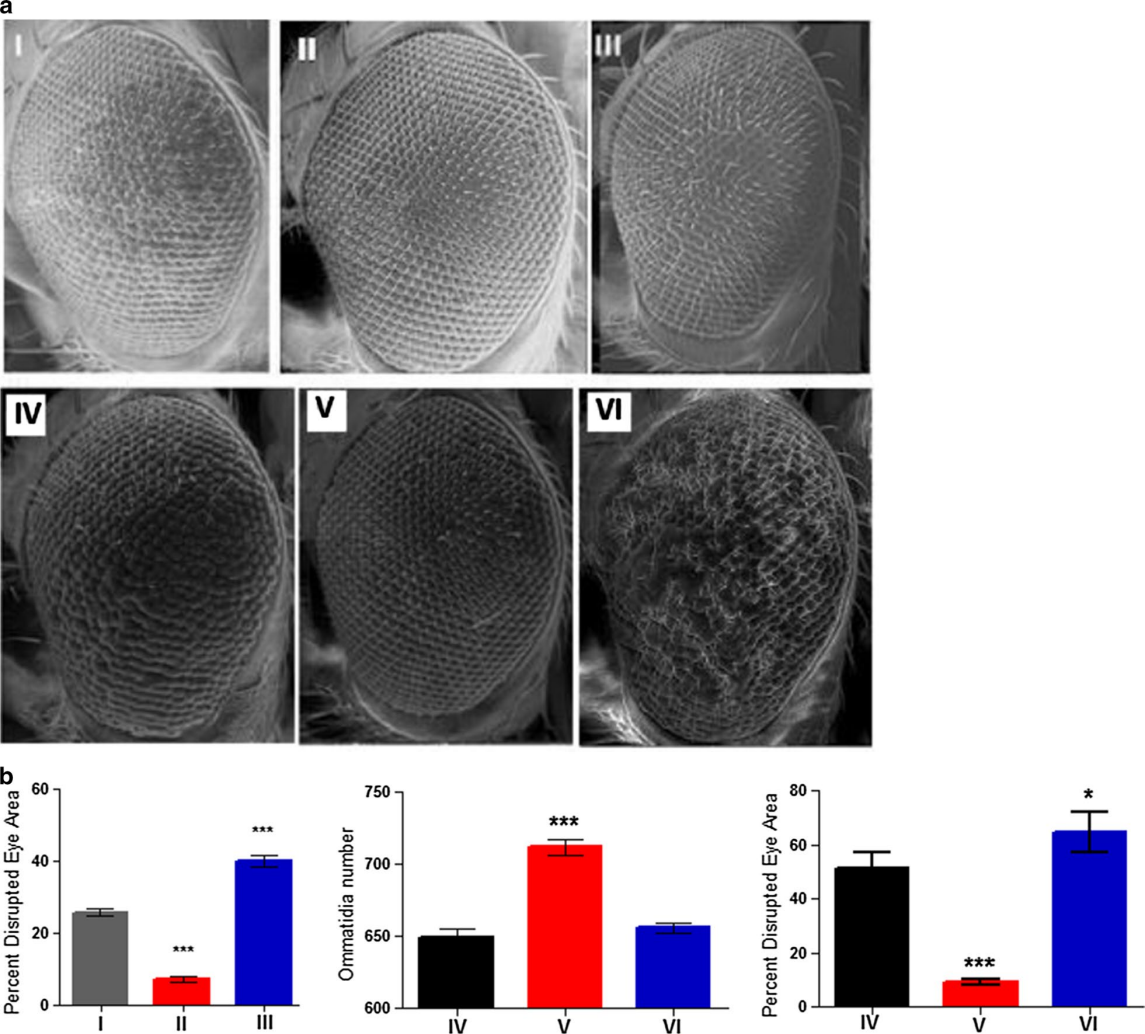
*Buffy* suppresses the *α*-*synuclein*-induced developmental defects in the eye. **a** Scanning electron micrographs when *Buffy* is overexpressed or inhibited in the eye with the eye-specific *GMR*-*Gal4* transgene; (*I*) *GMR*-*Gal4/UAS*-*lacZ;* (*II*) *GMR*-*Gal4/UAS*-*Buffy;* (*III*) *GMR*-*Gal4/UAS*-*Buffy*-*RNAi* and when co-expressed with *α*-*synuclein*; (*IV*) *UAS*-*α*-*synuclein; GMR*-*Gal4/UAS*-*lacZ;* (*V*) *UAS*-*α*-*synuclein; GMR*-*Gal4/UAS*-*Buffy;* and (*VI*) *UAS*-*α*-*synuclein; GMR*-*Gal4/UAS*-*Buffy*-*RNAi.* **b** Biometric analysis showed a significant difference in the disrupted area of the eye when *Buffy* was inhibited in the developing eye (*I*–*III*). Biometric analysis shows a marked difference when *Buffy* is inhibited in an *α*-*synuclein* expressing background (*IV*–*VI*) with decreased ommatidia number and highly degenerated ommatidial array whereas when *Buffy* is overexpressed in the *α*-*synuclein* background, there is a rescue of the *α*-*synuclein*-induced phenotypes as determined by one-way ANOVA and Dunnett’s multiple comparison test (P < 0.05 and 95 % CI), *error bars* indicate the SEM, *asterisks* represents statistically significant result and *n* = 10

The expression of *α*-*synuclein* in the developing eye results in developmental defects. When *Buffy* is overexpressed in the developing eye, developmental defects resulting from *GMR*-*Gal4* (Fig. 4b, I) and from *GMR*-*Gal4* and *α*-*synuclein* expression (Fig. 4b, IV) are suppressed. The disruption of the ommatidial array is restored to control levels in both cases (Fig. 4b). These results suggest that overexpression of *Buffy* is able to counteract the toxic effects of *α*-*synuclein* in the developing eye in addition to the effects of *GMR*-*Gal4*.

## Discussion

The recapitulation of PD-like symptoms in *Drosophila melanogaster* and especially the age-dependent loss of climbing ability led to the investigation of gene products that could suppress this phenotype [12, 13, 54]. Mitochondrial dysfunction as a result of α-synuclein accumulation has been implicated in PD pathogenesis and, thus, we have investigated the consequences of the overexpression of the Drosophila *Bcl*-*2* homologue *Buffy.* The analysis of climbing over the lifespan of the flies has been applied to determine the role of the various gene products in rescuing the *α*-*synuclein*-induced phenotypes [12, 54–56]. This assay allows for scoring of flies based on their loss of climbing ability and is a key indicator of the effect the overexpressed gene has on the phenotype.

The *α*-*synuclein*-expressing models of PD in Drosophila show little difference in lifespan between the control and wild type, A53T and A30P *α*-*synuclein* flies [12]. In our study, when *Buffy* is overexpressed in the DA neurons under the control of the *Ddc*-*Gal4* driver, there is a significant increase in their longevity. This may be partly due to defects in mitochondrial complex I function, the pro-survival *Buffy* likely plays a mitochondrial protective role to increase longevity. The inhibition of *Buffy* in the DA neurons resulted in a marked decrease in survival. This inhibition of *Buffy* is sufficient to negate its protective role and thus promote cell death as has recently been shown by Clavier and colleagues [57]. Thus, the pro-survival properties of Buffy are evident.

Locomotor dysfunction is one of the associated symptoms of PD, the *α*-*synuclein*-expressing model demonstrated a clear age-dependent loss in climbing ability [12]. When we overexpressed *Buffy* in the DA neurons under the control of *Ddc*-*Gal4,* the flies produced a climbing index significantly different from that of control flies. The *Buffy* flies lost the climbing ability later than the control flies and is likely due to the protective role that Buffy confers to the mitochondria. In contrast, the inhibition of *Buffy* results in a highly compromised climbing ability when compared to the controls. The degree of locomotor dysfunction seemed to be similar to that observed when *α*-*synuclein* is overexpressed in DA neurons. Taken together, these results would indicate an early protective role for *Buffy* in the DA neurons even in the absence of induced cellular stress.

The inhibition of *Buffy* in the DA neurons of the *α*-*synuclein*-induced PD model significantly decreased lifespan, indicating that low levels of Buffy compromise the health of DA neurons. When *Buffy* was overexpressed along with *α*-*synuclein*, there was a marked improvement in the climbing ability of these flies. These results suggest that overexpressing *Buffy* in the DA neurons counteracts the *α*-*synuclein*-induced phenotype of locomotor dysfunction over their lifespan. The *Buffy* loss-of-function flies displayed a reduced climbing ability compared to the control flies. Therefore, expression of the pro-survival *Buffy* can rescue the *α*-*synuclein*-dependent model of PD from climbing dysfunction.

Directed overexpression of *Buffy* in the developing eye rescues the roughened eye phenotypes caused by *Gal4* and *α*-*synuclein* expression, whereas the inhibition of *Buffy* results in a more disrupted ommatidial array. This indicates that elevated levels of *Buffy* in the developing eye offers protection from toxic protein insults to normalize cellular differentiation, neurogenesis and cell survival.

## Conclusions

*Buffy* inhibition results in shortened lifespan and impaired locomotor function and represents a novel model of PD in *Drosophila melanogaster*. The overexpression of *Buffy* improves healthspan and counteracts the effects of *α*-*synuclein* expression to demonstrate its protective function. Further studies are required to fully elucidate how *Buffy* may interact with the other PD genes, and how these interactions fit into the regulation of mitochondrial integrity by the Bcl-2 proteins.

## Methods

### Drosophila media and culture

Stocks and crosses were maintained on a standard medium containing cornmeal, molasses, yeast, agar, water and treated with propionic acid and methylparaben. Seven millilitre aliquots of media was poured into vials, allowed to solidify, and refrigerated at 4–6 °C. Stocks were maintained on solid media for two to 3 weeks before transfer onto new media to re-culture. Stocks were kept at room temperature (22 ± 2 °C) while crosses and experiments were carried out at 25 and 29 °C.

### Drosophila stocks and derivative lines

*UAS*-*Buffy* [52] was generously provided by Dr. Leonie Quinn (University of Melbourne), *UAS*-*α*-*synuclein* [12] was provided by Dr. M. Feany of Harvard Medical School and Dr. J. Hirsch (University of Virginia) provided *Ddc*-*Gal4* flies [58]. *UAS*-*Buffy*-*RNA*_*i*_ (w[*]; P{w[+mC] = UAS-Buffy.RNAi}3), *GMR*-*Gal4* [59] and *UAS*-*lacZ* flies were obtained from the Bloomington Drosophila Stock Center at Indiana University. The *UAS*-*α*-*synuclein/CyO; Ddc*-*Gal4/TM3* was generated using standard homologous recombination methods and was used to overexpress *α*-*synuclein* in the dopaminergic neurons using the *dopa decarboxylase* (*Ddc*) transgene. The *UAS*-*α*-*synuclein/CyO; GMR*-*Gal4* line was used to overexpress *α*-*synuclein* in the developing eye using the *Glass Multiple Reporter* (*GMR*) elements. PCR reactions and gel electrophoresis were used for analysis of recombination events.

### Ageing assay

Several single vial matings of five females and three males of each genotype were made and a cohort of critical class male flies were collected upon eclosion. At least two hundred flies were aged per genotype at a density of ≤20 flies per vial on fresh media which was replenished every other day to avoid crowding. Flies were observed and scored every 2 days for the presence of deceased adults. Flies were considered dead when they did not display any movement upon agitation [60]. Longevity data was analysed using the GraphPad Prism version 5.04 and survival curves were compared using the log-rank (Mantel-Cox) test. Significance was determined at 95 %, at a P value less than or equal to 0.05 with Bonferroni correction.

### Climbing assay

A cohort of critical class male flies was collected upon eclosion and scored for their ability to climb over their lifetime [56, 61]. Every 7 days, 50 males from every genotype were assayed for their ability to climb 10 cm in 10 s in a clean climbing apparatus in ten repetitions. Analysis was performed using the GraphPad Prism version 5.04 and climbing curves were fitted using non-linear regression and compared using 95 % confidence interval with a 0.05 P value.

### Scanning electron microscopy of the Drosophila eye

Several single vial matings were made at 29 °C and a cohort of adult male flies collected upon eclosion and aged for 3 days before being frozen at −80 °C. Whole flies were mounted on scanning electron microscope stubs, desiccated overnight and photographed with a FEI Mineral Liberation Analyzer 650F scanning electron microscope. For each cross at least 10 eye images were analysed using the National Institutes of Health (NIH) ImageJ software [62] and biometric analysis performed using GraphPad Prism version 5.04. The percent area of eye disruption was calculated as previously described [63].

## Abbreviations

BH: Bcl-2 homology
Bcl-2: B cell lymphoma 2
DA: dopaminergic
Ddc: dopa decarboxylase
PD: Parkinson disease
RNAi: ribonucleic acid interference
SEM: standard error of the mean
